# Elevated glucose is associated with hemorrhagic transformation after mechanical thrombectomy in acute ischemic stroke patients with severe pretreatment hypoperfusion

**DOI:** 10.1038/s41598-020-67448-x

**Published:** 2020-06-29

**Authors:** Carlos Laredo, Arturo Renú, Laura Llull, Raúl Tudela, Antonio López-Rueda, Xabier Urra, Napoleón G. Macías, Salvatore Rudilosso, Víctor Obach, Sergio Amaro, Ángel Chamorro

**Affiliations:** 1 Comprehensive Stroke Center, Department of Neuroscience, Hospital Clinic, University of Barcelona and August Pi i Sunyer Biomedical Research Institute (IDIBAPS), Villarroel 170, 08036 Barcelona, Spain; 2 CIBER de Bioingeniería, Biomateriales y Nanomedicina (CIBER-BBN), Group of Biomedical Imaging of the University of Barcelona, Barcelona, Spain; 3 Radiology Department, Hospital Clinic, Barcelona, Spain

**Keywords:** Neurology, Stroke

## Abstract

Several pretreatment variables such as elevated glucose and hypoperfusion severity are related to brain hemorrhage after endovascular treatment of acute stroke. We evaluated whether elevated glucose and severe hypoperfusion have synergistic effects in the promotion of parenchymal hemorrhage (PH) after mechanical thrombectomy (MT). We included 258 patients MT-treated who had a pretreatment computed tomography perfusion (CTP) and a post-treatment follow-up MRI. Severe hypoperfusion was defined as regions with cerebral blood volume (CBV) values < 2.5% of normal brain [very-low CBV (VLCBV)-regions]. Median baseline glucose levels were 119 (IQR = 105–141) mg/dL. Thirty-nine (15%) patients had pretreatment VLCBV-regions, and 42 (16%) developed a PH after MT. In adjusted models, pretreatment glucose levels interacted significantly with VLCBV on the prediction of PH (p-interaction = 0.011). In patients with VLCBV-regions, higher glucose was significantly associated with PH (adjusted-OR = 3.15; 95% CI = 1.08–9.19, *p* = 0.036), whereas this association was not significant in patients without VLCBV-regions. CBV values measured at pretreatment CTP in coregistered regions that developed PH or infarct at follow-up were not correlated with pretreatment glucose levels, thus suggesting the existence of alternative deleterious mechanisms other than direct glucose-driven hemodynamic impairments. Overall, these results suggest that both severe hypoperfusion and glucose levels should be considered in the evaluation of adjunctive neuroprotective strategies.

## Introduction

Mechanical thrombectomy (MT) is the most effective treatment for stroke patients with acute large-vessel occlusions in the carotid territory^1,2^. However, about half of MT-treated patients do not achieve an adequate clinical recovery at follow-up even despite complete recanalization^1,2^. One of the pathophysiological processes that has been implicated in the lack of response to recanalization therapies is the risk of hemorrhagic transformation due to blood–brain barrier disruption^3,4^. Indeed, among patients with anterior circulation stroke undergoing endovascular therapy, the presence of hemorrhagic transformation in follow-up neuroimaging, especially in the form of parenchymal hematomas (PH), has been associated with poor long term functional outcome^5,6^.

The presence of PH in post-treatment neuroimaging occurs in 5 to 16% of acute stroke patients treated with MT and has been related with several pretreatment variables including severe hypoperfusion and hyperglycemia^7,8^. The presence of brain regions with very low cerebral blood volume (VLCBV) in pretreatment computed tomography perfusion (CTP) has been consistently associated with the risk of hemorrhagic transformation although the sensitivity and positive predictive values of risk estimations are relatively low compared with its high specificity and negative predictive values^9–11^. Accordingly, severe hypoperfusion might not be a sufficient risk factor for the occurrence of hemorrhagic transformation and additional concurrent susceptibility conditions might be needed, such as pretreatment hyperglycemia. The presence of hyperglycemia at stroke onset has been associated with a higher risk of hemorrhagic transformation after reperfusion therapies as well as with significant reductions on the benefits of MT^4,12,13^. The potential mechanisms contributing to exacerbated neurovascular injury and poor outcomes are multifactorial and include augmented oxidative and nitrosative stress, tissue acidosis, mitochondrial dysfunction, thromboinflammation and impairment of cerebral perfusion, among others^14,15^. However, in preclinical models the interaction between hyperglycemia and reduced cerebral blood volume or blood flow during ischemia and at reperfusion is controversial^16–23^.

Herein, we aimed to evaluate whether elevated glucose levels modify the association between severe hypoperfusion and hemorrhagic transformation after MT and to describe whether this association might be explained by glucose-driven hemodynamic impairments before reperfusion.

## Methods

### Patients

Patients were part of a prospectively collected clinical registry of acute ischemic stroke patients treated with reperfusion therapies in a single Comprehensive Stroke Center. The study population included consecutive patients with occlusions in the carotid territory treated with endovascular recanalization therapy between March 2010 and December 2017. Additional inclusion criteria for this analysis were: 1/the availability of a technically adequate pretreatment whole-brain CTP scan, and 2/the availability of a post-treatment follow-up MRI for evaluating the development of hemorrhagic transformation. A total of 258 patients fulfilled these criteria and were finally included in the study (Supplementary Fig. S1 online). MT was performed according to contemporary guidelines in patients with a proximal artery occlusion on CT angiography and a pretreatment ASPECTS equal or higher than 6, as previously reported^11^. Final vessel patency was graded on digital subtraction angiography (DSA) at the end of MT according to the modified Thrombolysis in Cerebral Infarction (TICI) score and successful recanalization was defined as a grade 2b-3. The study protocol was approved by the local Clinical Research Ethics Committee of Hospital Clinic de Barcelona (registration number HCB/2019/0805) under the requirements of Spanish legislation in the field of biomedical research, the protection of personal data (15/1999) and the standards of Good Clinical Practice, as well as with the 1964 Helsinki declaration and its later amendments or comparable ethical standards. Participants in the study consented for storage of their data in a local database for the purpose of research that was declared into a Web-based registry that satisfied all legal requirements for protection of personal data, for monitoring by the Catalan Health Department.

Patients were admitted into a Stroke Unit and the Trial of Org 10,172 in Acute Stroke Treatment (TOAST) criteria was used to classify all qualifying strokes after diagnostic workup. Clinical data was prospectively collected including: demographics, laboratory tests, risk factors, concomitant therapies, neuroimaging, clinical course and functional outcome. Neurological status was monitored with the National Institutes of Health Stroke Scale (NIHSS) score and functional outcome was quantified using the modified Rankin Scale (mRS) score at 3 months^3^.

### CTP imaging analysis

Patients were scanned using a SIEMENS Somatom Definition Flash 128-section dual-source multidetector scanner (Siemens Healthineers, Erlangen, Germany), with a 98 mm z-coverage and a total acquisition time of 60 s (31 time points), as previously described^11^. The imaging protocol included a baseline multimodal whole-brain CT scan, which included a Non-Contrast CT (NCCT), a CT angiography and a CTP. Pretreatment ASPECTS was assessed on NCCT, and good collaterals were defined as collateral supply filling > 50% of the occluded arterial territory^24^. CTP maps were calculated by commercial software MIStar (Apollo Medical Imaging Technology, Melbourne, Australia) using a model-free singular value decomposition algorithm with a delay and dispersion correction. The software generates cerebral blood flow (CBF), cerebral blood volume (CBV), mean transit time (MTT) and Delay Time (DT) maps. An image processing pipeline using in-house fully-automated software running in Matlab (v.2017b, Mathworks, Natick, MA) was developed in order to extract perfusion volumes. An absolute threshold of 3 s was selected on the DT map to obtain the hypoperfused tissue (perfusion lesion)^11^. Ischemic core was extracted on CBF maps after applying a threshold of relative CBF < 30%. VLCBV was defined as values on CBV maps lower than 2.5% of normal brain in the contralateral hemisphere with a volume of at least 1.5 mL^11^.

### Follow-up MRI

After MT, a follow-up MRI was performed within a median of 41 h (IQR 26–69 h) of hospital admission. The MRI included diffusion-weighted images and Gradient-Echo T2*-weighted sequences. The bleeding complications were scored on follow-up Gradient-Echo T2*-weighted sequences by 2 experienced stroke neurologists (A.R. and S.A.) according to the European Cooperative Acute Stroke Study criteria as hemorrhagic infarction (HI) and PH type 1 and type 2^25^. In brief, PH1 was defined as bleeding ≤ 30% of the infarcted area with mild space-occupying effect, and PH2 as bleeding > 30% of the infarcted area, with space-occupying effect. Symptomatic intracranial hemorrhage was defined as any PHs associated with an increment of at least 4 points in the NIHSS score. Investigators blinded to clinical data and baseline CTP analysis evaluated in consensus the post-treatment imaging studies. To perform a CTP-MRI regions of interest analysis each CTP map was coregistered to the corresponding follow-up DWI using a rigid coregistration protocol (6-degrees of freedom) implemented with statistical parametric mapping (SPM12, Functional Imaging Laboratory, University College London, London, UK). An extended description of the imaging methods can be found in the Suplementary methods.

### Statistics

Continuous variables were reported as mean (standard deviation, SD) or median (interquartile range, IQR) and were compared with the Student t test, ANOVA, Mann–Whitney, or Kruskal–Wallis tests as appropriate. Categorical variables were compared with the χ^2^ and Fisher exact tests. Univariate logistic regression models were used to assess the association between VLCBV-regions, glucose levels and their interaction on the risk of PH and with other predefined clinical endpoints. Multivariate logistic regression models were used to adjust the estimations for the effect of confounders based on exploratory analysis (*p* < 0.05 on univariate analysis) and avoidance of collinearity. A backwards-stepwise procedure was implemented to reach the final models constructed for assessing the role of pretreatment glucose levels on the risk of PH in subgroups defined by VLCBV regions in order to avoid overfitting. The Hosmer–Lemeshow goodness-of-fit statistic was used to assess final model fit. Receiver-operating characteristic (ROC) curve analysis was performed to determine the most accurate cutoff point (Youden's index) of pretreatment glucose levels for the prediction of PH. To evaluate the relationship between glucose levels and CBV values a set of regression models were used including linear, logarithmic, quadratic and cubic curve estimations. The analyses were performed using SPSS Version 22.0 and the level of significance was established at the 0.05 level (2-sided).

## Results

### Baseline traits of the included population according to PH occurrence

Overall, 258 patients with a median (IQR) NIHSS 17 (10–20) at admission and treated with MT within a median (IQR) of 231 (161–334) minutes from stroke onset were included in the study. Median (IQR) baseline glucose levels were 119 (105–141) mg/dL (6.6 mmol/L [5.8–7.8] mmol/L). Thirty-nine (15%) patients had pretreatment VLCBV-regions, and 42 (16%) developed a PH in follow-up neuroimaging [30 (12%) had PH1 and 12 (5%) had PH2]. Histograms showing the distribution of pretreatment glucose levels and the volume of regions with VLCBV are shown in the Supplementary Figure S2 online, and the baseline traits of the population according to the presence of VLCBV regions are shown in Supplementary Table 1. Table 1 provides descriptive data on baseline clinical and radiological variables associated with the occurrence of PH. In univariate analysis, PH was associated with a higher baseline stroke severity (NIHSS and ASPECTS score), a larger volume of ischemic tissue with VLCBV, a higher proportion of VLCBV regions, cardioembolic stroke etiology, as well as longer times from stroke onset to CTP acquisition, MT onset and successful recanalization. The presence of PH at follow-up was associated with increased odds for shifting to worse mRS score categories (OR 1.89, 95% CI 1.08–3.30, p = 0.025).

**Table 1:** Demographics, baseline and procedure related variables according to the occurrence of parenchymal hematoma.

	No PH N = 216	PH N = 42	*p*
Age (years), median (IQR)	72 (61–80)	67 (59–79)	0.481
Males, n (%)	106 (49)	26 (62)	0.128
Hypertension, n (%)	121 (56)	26 (62)	0.481
Diabetes, n (%)	31 (14)	5 (12)	0.675
Dyslipidemia, n (%)	83 (38)	15 (36)	0.740
Atrial Fibrillation, n (%)	59 (27)	16 (38)	0.159
Previous Antithrombotic treatment, n (%)	84 (39)	20 (48)	0.291
Baseline SBP (mmHg), median (IQR)	140 (125–158)	139 (125–158)	0.793
Glucose (mg/dL), median (IQR)	119 (105–141)	119 (107–143)	0.779
NIHSS at admission, median (IQR)	17 (10–20)	19 (14–21)	0.045
Ischemic core on CTP (mL), median (IQR)	19 (7–34)	18 (11–45)	0.273
Hypoperfused tissue on CTP (mL), median (IQR)	135 (98–186)	144 (99–198)	0.633
VLCBV (mL), median (IQR)	0.03 (0–0.31)	0.34 (0.03–2.07)	0.002
VLCBV regions, n (%)	26 (12)	13 (31)	0.002
Good collaterals, n (%)	158 (73)	27 (64)	0.243
Alteplase + MT, n (%)	116 (54)	25 (60)	0.488
Time to CTP (min), md (IQR)	148 (77–252)	242 (141–339)	0.001
Time to MT onset (min), md (IQR)	231 (161–334)	327 (230–425)	0.001
Recanalization (yes), n (%)	170 (79)	34 (81)	0.743
Time to recanalization (min), median (IQR)	270 (202–375)	359 (303–479)	0.001
Recanalization groups			0.002
Recanalization < 4.5 h, n (%)	86 (40)	6 (14)	
Recanalization > 4.5 h, n (%)	84 (39)	28 (67)	
No rec, n (%)	46 (21)	8 (19)	
Time to MRI (hours), md (IQR)	40 (26–65)	43 (24–69)	0.589
Cardioembolic origin, n (%)	103 (48)	27 (64)	0.049
Location of the occlusion			0.436
Tandem occlusions, n (%)	33 (15)	7 (17)	
ICA-T or M1, n (%)	169 (78)	30 (71)	
M2, n (%)	14 (7)	5 (12)	
Follow-up clinical variables			
Symptomatic ICH, n (%)	1 (1)	7 (17)	< 0.001
mRS at 90 days, median (IQR)	2 (1–3)	3 (2–4)	0.019

*ASPECTS* Alberta Stroke Program Early CT Score, *CTP* computed tomographic perfusion, *ET* endovascular therapy, *ICA-T* internal carotid artery, *ICH* intracranial hemorrhage, *mRS* modified Rankin Scale, *MT* mechanical thrombectomy, *NIHSS* National Institutes of Health Stroke Scale, *PH* parenchymal hematoma, *SBP* systolic blood pressure, *VLCBV* very low cerebral blood volume.

### Predictors of PH in the whole population: multivariate analysis

In multivariate analyses, both the volume of ischemic tissue with VLCBV values and the presence of VLCBV regions were associated with an increased risk of PH (Table 2). Overall, the sensitivity, specificity, positive predictive value and negative predictive value of the presence of VLCBV regions for predicting the occurrence of PH were 31%, 88%, 33% and 87%, respectively. Additional variables that remained associated with PH were successful recanalization beyond 4.5 h from stroke onset and cardioembolic stroke etiology.

**Table 2:** Predictors of parenchymal hematoma: multivariate analysis.

PH at follow-up MRI	OR (95% CI); *p*	OR (95% CI); *p*
	Model A	Model B
VLCBV regions	2.816 (1.146–6.918), *p* = 0.024	1.592 (1.133–2.237), *p* = 0.007
Pretreatment glucose (per IQR)	1.049 (0.755–1.456), *p* = 0.777	1.072 (0.771–1.491), *p* = 0.678
Baseline NIHSS (per IQR)	1.337 (0.948–1.887), *p* = 0.098	1.260 (0.885–1.795), *p* = 0.200
Rescue MT (vs primary)	1.395 (0.668–2.916), *p* = 0.376	1.415 (0.674–2.971), *p* = 0.359
Recanalization status		
> 4′5 h from stroke onset (vs < 4′5 h)	6.656 (2.444–18.130), *p* < 0.001	7.552 (2.745–20.779), *p* < 0.001
No recanalization (vs < 4′5 h)	2.848 (0.867–9.351), *p* = 0.084	2.810 (0.865–9.132), *p* = 0.086
Cardioembolic etiology (vs no)	2.584 (1.197–5.577), *p* = 0.016	2.812 (1.285–6.153), *p* = 0.010
Sex (females vs males)	0.500 (0.228–1.097), *p* = 0.084	0.522 (0.237–1.150), *p* = 0.107
Good collaterals (vs poor)	1.256 (0.550–2.867), *p* = 0.588	1.369 (0.599–3.129), *p* = 0.456

Very low cerebral blood volume (VLCBV) was included as a dichotomic variable (yes vs no) in model A, and as a continuous quantitative variable (estimations per IQR of VLCBV increase) in model B. MT: Mechanical thrombectomy; NIHSS: National Institutes of Health Stroke Scale; PH: parenchymal hematoma. The Hosmer–Lemeshow test showed an adequate goodness-of-fit of the final models (Model A: X2 = 4.028, *p* = 0.855; Model B: X2 = 9.329, *p* = 0.315), and the models classified correctly a total of 84% (Model A) and 85% (Model B) of cases.

### Glucose, VLCBV and risk of PH

**Figure 1:**
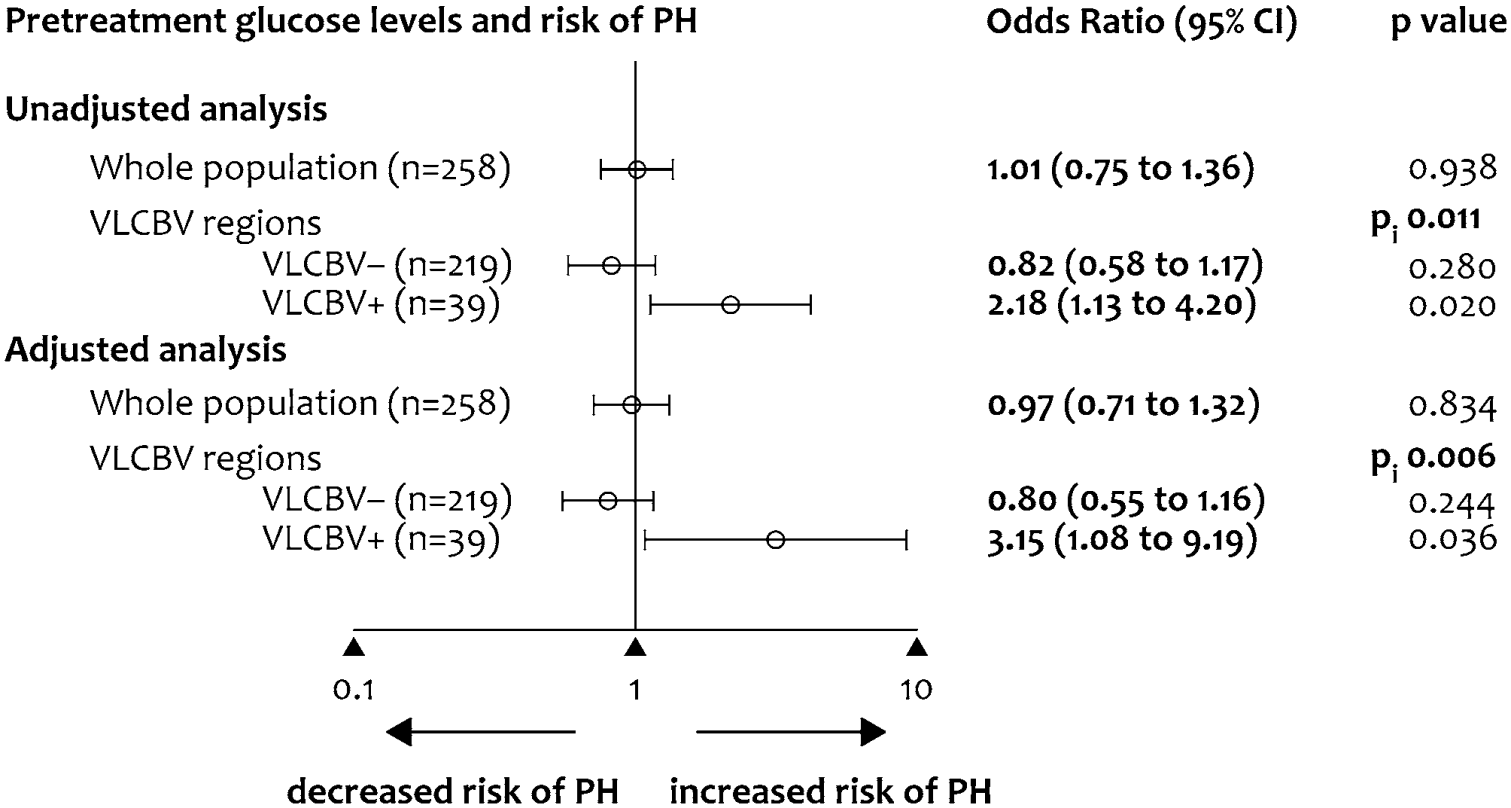
Binary regression models for predicting the occurrence of parenchymal hematoma (PH) in follow-up MRI. Pi is the p value for the interaction between pretreatment glucose levels and the presence of very low cerebral blood volume (VLCBV) regions for the prediction of PH. Data are OR and 95% CI per IQR increase in glucose levels obtained by unadjusted models and in models adjusted for pretreatment NIHSS, recanalization and cardioembolic stroke etiology (see Supplemental Table 3 for full models).

In the whole population, glucose levels were not associated with the presence of PH. However, pretreatment glucose levels interacted significantly with VLCBV for the prediction of PH (p = 0.011). In patients with VLCBV-regions (see Supplementary Table 2 for subgroup descriptive variables), glucose levels were associated with an increased risk of PH (OR = 2.18 per IQR increase in glucose levels; 95% CI = 1.13–4.200, p = 0.020), whereas this association was not significant in patients without VLCBV-regions. The direction of these observations remained unchanged in multivariate models adjusted for the effect of confounders (Fig. 1). Neither glucose nor VLCBV interacted significantly with mTICI score at the end of MT (p = 0.622 and p = 0.389, respectively) or with time from stroke onset to successful recanalization on the prediction of PH (p = 0.202 and p = 0.777, respectively).

**Figure 2:**
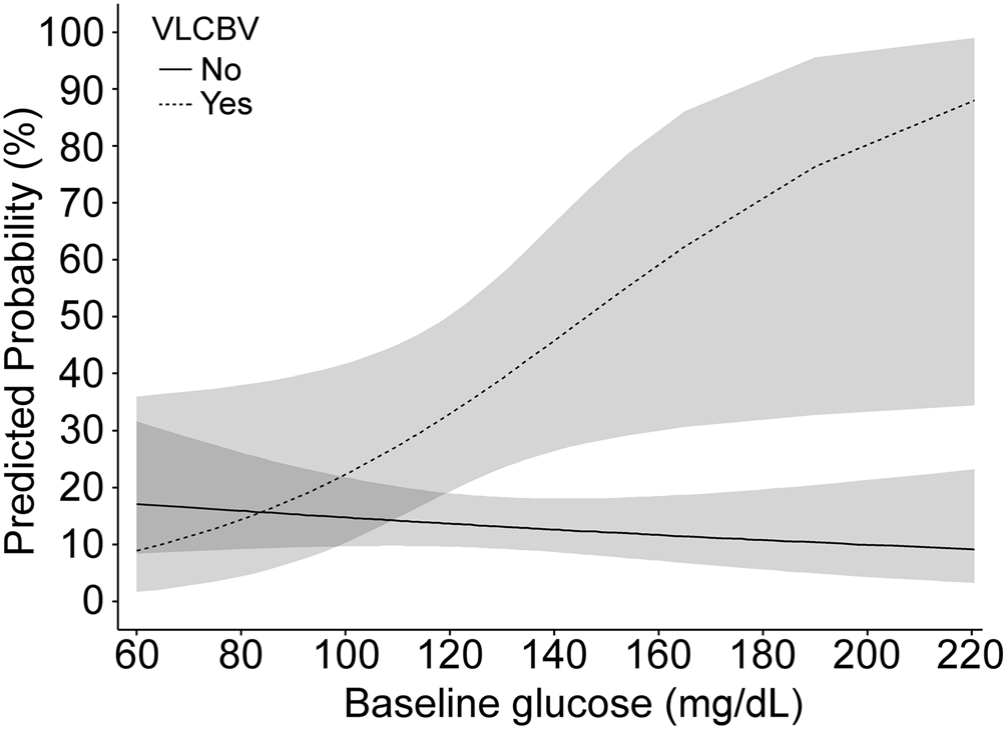
Predicted probability of parenchymal hematoma (PH) at follow-up MRI. Predicted probability of parenchymal hematoma (PH) at follow-up MRI by pretreatment glucose levels according to subgroups defined by the presence or absence of very low cerebral blood volume (VLCBV) regions. Dashed lines show the 95% CI. For graphical purposes, pretreatment glucose was analyzed as a continuous variable. The predicted probabilities were obtained in models adjusted for baseline NIHSS, reperfusion treatment modality, recanalization and stroke etiology.

Figure 2 shows the adjusted predicted probabilities of the occurrence of a PH according to pretreatment glucose levels and VLCBV volume. In adjusted models, per IQR increase in glucose levels, the predicted probability of PH increased in 75% in patients with VLCBV regions (aOR = 3.07, 95% CI = 1.04–9.10, p = 0.043). According to ROC analysis, in the subset of patients with VLCBV regions the cutoff of pretreatment glucose levels with best accuracy for predicting the occurrence of PH was 116 mg/dL (6.4 mmol/L; sensitivity 69%, specificity 65%, positive predictive value 50% and negative predictive value 81%). According to this categorization, a total of 18 patients from 39 of those with VLCBV regions had elevated glucose levels. The observed rate of PH increased steadily from 13% (29 of 219) in patients without VLCBV regions, 19% (4 of 21) in those with VLCBV regions and low glucose levels (≤ 116 mg/dL, ≤ 6.4 mmol/L) and 50% (9 of 18) in patients with VLCBV regions and high glucose levels (> 116 mg/dL, > 6.4 mmol/L) (*p* for trend < 0.001).

### Hemodynamic correlates of PH according to pretreatment glucose levels: CTP-MRI coregistered regions of interest analysis

**Figure 3:**
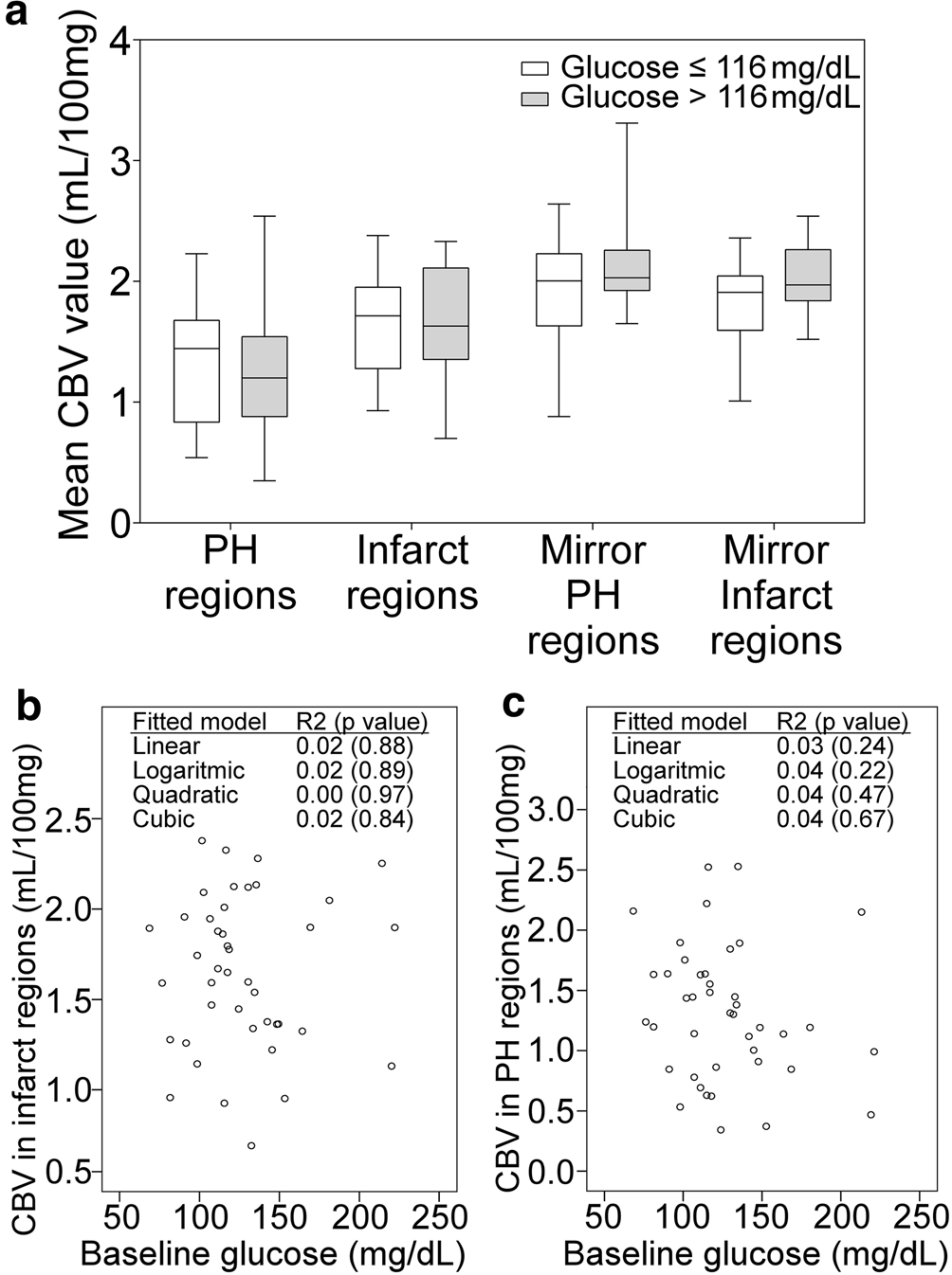
Cerebral blood volume values measured at pretreatment CTP in coregistered brain regions that developed PH or infarct at follow-up. (**a)** Box-whisker plots of cerebral blood volume (CBV) values extracted from pretreatment computed tomographic perfusion (CTP) in coregistered regions that developed infarct or parenchymal hematoma (PH) at follow-up according to the presence or absence of pretreatment glucose levels higher than 116 mg/dL (6.4 mmol/L). (**b**, **c)** Association between baseline glucose levels and CBV values extracted from pretreatment CTP in coregistered regions that developed infarct (**b**) or PH (**c**) at follow-up MRI.

As shown in Fig. 3A, brain regions that developed PH or infarct at follow-up had lower CBV values in pretreatment CTP compared with mirror regions of the unaffected hemisphere. In addition, CBV values measured at baseline in coregistered brain regions that developed PH or infarct at follow-up were not correlated with pretreatment glucose levels and were similar in patients with levels higher or lower than 116 mg/dL (6.4 mmol/L), as illustrated in Fig. 3B, C. Finally, the quality of reperfusion measured with the mTICI score at the end of MT was not associated with pretreatment glucose levels (*p* = 0.952).

### Glucose, VLCBV and clinical outcome

The association of pretreatment glucose levels, VLCBV regions and their interactions with symptomatic hemorrhagic transformation, mRS at day 90 and mortality are shown in Table 3. Of note, the interaction between pretreatment glucose levels and the presence of VLCBV regions on the prediction of these clinical outcomes was not significant.

**Table 3:** Association of pretreatment glucose levels and VLCBV with clinical endpoints.

	Pretreatment glucose (per IQR increase)	VLCBV regions (yes vs no)	Pi
mRS shift at 90 days	1.310 (1.091–1.587), p = 0.006	1.587 (0.887–2.838), p = 0.120	0.562
Symptomatic ICH	0.804 (0.424–1.525), p = 0.504	0.797 (0.095–6.663), p = 0.834	0.998
Mortality at 90 days	0.990 (0.612–1.603), p = 0.968	0.932 (0.200–4.338), p = 0.929	0.751

Odds ratios, 95% confidence intervals and p values per IQR increase in pretreatment glucose levels (second column) and according to the presence of very low cerebral blood volume (VLCBV) regions (third column) obtained through unadjusted logistic regression analysis. Pi values (fourth column) are the p values for the interaction between pretreatment glucose levels and the presence of VLCBV regions on each of the evaluated clinical outcomes. *ICH* intracranial hemorrhage, *mRS* modified Rankin Scale.

## Discussion

In this study, we implemented a comprehensive CTP study to evaluate whether elevated glucose levels modified the association between severe hypoperfusion and PH after MT. In this cohort, elevated pretreatment glucose levels were associated with an increased risk of PH after MT in subjects with severe pretreatment hypoperfusion. Moreover, glucose levels were not associated with reduced CBV at pretreatment CTP in coregistered brain regions that developed PH or infarct at follow-up. Overall, these results suggest the existence of synergistic deleterious effects of severe pretreatment hypoperfusion and elevated glucose and give support to their consideration in the evaluation of adjunctive neuroprotective strategies.

In agreement with previous studies, the presence of regions with severe hypoperfusion was significantly associated with an increased risk of PH at follow-up neuroimaging^9–11,26–29^. Beyond the severity of brain ischemia, a number of additional variables have been also associated with an increased risk of hemorrhagic transformation after MT, including an increased NIHSS score, poor collaterals, antiplatelet use, atrial fibrillation, older age or pretreatment hyperglycemia, among others^6^. As a novel finding of this study, we found that pretreatment glucose levels interacted significantly with severe hypoperfusion on the prediction of PH. Thus, in patients with regions of VLCBV, higher pretreatment glucose levels increased significantly the risk of PH, while this association was not significant in patients without VLCBV regions. Indeed, about 1 in 5 patients with VLCBV regions and low pretreatment glucose levels at baseline developed a PH in follow-up neuroimaging, whereas this rate increased to 1 in 2 patients with concurrent high glucose levels. The consideration of pretreatment glucose in addition to the presence of VLCBV regions resulted in higher positive predictive value for the prediction of PH in comparison with VLCBV alone. From a practical point of view, the combination of these biomarkers could be useful in the evaluation of strategies aimed to protect the blood–brain barrier in this patient population as patients with regions of severe ischemia and elevated pretreatment glucose levels may represent a target population for the early implementation of preventive strategies. These strategies could theoretically include a tighter control of blood pressure, the avoidance of early post-treatment aggressive antithrombotic therapy or the addition of adjunctive neuroprotective or vasculoprotective therapies. Of these potential treatments, the implementation of strategies focused in lowering glucose concentrations and achieving a tight glucose control in the early acute phase have been repeatedly unsuccessful in humans regardless of preventing lactic acidosis^30–32^. Contrarily, preliminary data from preclinical and clinical studies support that the enhancement of antioxidant exposure in combination with reperfusion therapies could minimize the toxicity of hyperglycemia^33^. Importantly, given the strong clinical benefits of MT, the presence of regions of severe reductions of CBV in CTP and concurrent hyperglycemia should not preclude the treatment of patients otherwise eligible for receiving endovascular reperfusion therapies.

In experimental models of brain ischemia, hyperglycemia exacerbates ischemic brain injury by increasing infarct size, brain swelling and blood–brain barrier disruption^34–37^. Several preclinical studies have shown a direct association between induced hyperglycemia and poorer perfusion metrics during ischemia and after reperfusion whereas others were not able to identify such relationship^16–23^. In this study, pretreatment CBV values were not correlated with pretreatment glucose levels and were similar in patients with levels higher or lower than 116 mg/dL (6.4 mmol/L), regardless of the presence of successful recanalization. In addition, the quality of reperfusion obtained at the end of MT was not associated with pretreatment glucose levels, in agreement with findings from a recent meta-analysis that included pooled-data of the pivotal thrombectomy trials^13^. Overall, our results support the relevance of additional pathophysiological mechanisms beyond hyperacute glucose-driven disturbances in cerebral perfusion to explain the association between hyperglycemia and severe hypoperfusion with the risk of PH, such as enhanced thromboinflammatory mechanisms, increased blood–brain barrier disruption and worse cytotoxic injury, among others^15,36,37^.

The main strength of the study was the use of whole-brain CTP that allowed obtaining perfusion measures of most of the affected brain tissue. Moreover, patients were collected consecutively and managed following a homogeneous therapeutic protocol. The management of hyperglycemia in the acute phase followed the recommendations of contemporary guidelines recommending the administration of insulin in patients with glucose concentrations > 140 mg/dL (7.8 mmol/L). Furthermore, although the sensitivity of MRI to detect hemorrhagic transformation is higher than CT, we do not think this is likely to have significantly affected our result because the imaging modality used for the attribution of the main outcome variable (PH) was identical (gradient-recall echo MRI sequence) in all the study population thus avoiding biases related to the use of different modalities (CT or MRI). Nonetheless, the study has several limitations. First, the assessment of perfusion with dynamic CT in acute stroke is a static evaluation of a multifaceted and time-dependent process. Secondly, perfusion measures may be affected by multiple factors, including acquisition protocols, brain coverage and post-processing platforms, and therefore our results may apply to the protocol of acquisition and analysis that were employed in these series and may not be generalizable to other methodologies. In this study, we used the European Cooperative Acute Stroke Study criteria for qualifying the type of hemorrhagic transformations to allow direct comparisons with previously reported studies on the same matter. Further validation of these results using the new Heidelberg Bleeding Classification specially for assessing the effect of hyperglycemia and severe hypoperfusion on the risk of symptomatic hemorrhagic transformation is warranted^38^. An additional limitation includes the lack of information on the longitudinal course of glucose at follow up and the undocumented use of blood glucose lowering drugs. Finally, the results were not controlled by type of stroke onset (witnessed versus unwitnessed) or pre-stroke fasting status, as those variables were not registered. Of note, given the retrospective nature of the study no causality assumptions can be inferred from the obtained data.

## Conclusions

In summary, this study shows that elevated pretreatment glucose levels were associated with an increased risk of parenchymal hematoma after endovascular reperfusion therapy of acute ischemic stroke in patients with severe pretreatment hypoperfusion. Moreover, the link between pretreatment glucose levels and severe hypoperfusion was not explained by an association of glucose with reduced CBV during ischemia and prior to reperfusion, thus suggesting alternative deleterious mechanisms other than direct glucose-driven hemodynamic impairment. These two factors should be considered in the evaluation of adjunctive neuroprotective strategies aimed to protect the blood–brain barrier in addition to early and complete recanalization.

## Supplementary information

Supplementary information

## Data Availability

The datasets analyzed during the current study are available from the corresponding author on reasonable request.
