# Supplementary material for: Elevated glucose is associated with hemorrhagic transformation after mechanical thrombectomy in acute ischemic stroke patients with severe pretreatment hypoperfusion
Source: Sci Rep. 2020 Jun 29;10:10588. doi: 10.1038/s41598-020-67448-x (PMC7324383; doi:10.1038/s41598-020-67448-x)
Supplement: Supplementary file 1 — Supplementary information [file 41598_2020_67448_MOESM1_ESM.pdf]

Supplementary Information

Title: Elevated glucose is associated with hemorrhagic transformation after mechanical thrombectomy in patients with severe pretreatment hypoperfusion.

Authors: Carlos Laredo,[1] Arturo Renú,[1] Laura Llull,[1] Raúl Tudela,[2] Antonio Lopez-Rueda,[3] Xabier Urra,[1] Napoleón G Macías,[3] Salvatore Rudilosso,[1] Víctor Obach,[1] Sergio Amaro*,[1] Ángel Chamorro*.[1]

Affiliations

[1] Comprehensive Stroke Center, Department of Neuroscience, Hospital Clinic, University of Barcelona and August Pi i Sunyer Biomedical Research Institute (IDIBAPS), Barcelona, Spain.

[2] CIBER de Bioingeniería, Biomateriales y Nanomedicina (CIBER-BBN), Group of Biomedical Imaging of the University of Barcelona, Barcelona, Spain.

[3] Radiology Department, Hospital Clinic, Barcelona, Spain.

Corresponding authors*: Sergio Amaro and Angel Chamorro; Hospital Clinic, Villarroel 170, 08036 Barcelona, Spain. Email: samaro@clinic.cat and achamorro@clinic.cat; Tel.: + 34932275414; fax: +34932275783.

Supplementary methods

CTP imaging and follow-up MRI parameters

Patients were scanned using a SIEMENS Somatom Definition Flash 128-section dual-source multidetector scanner (Siemens Healthcare, Erlangen, Germany), with a 98mm z-coverage and a total acquisition time of 60s (31 time points). The imaging protocol included a baseline multimodal whole-brain CT scan, which included a Non-Contrast CT (NCCT) (140Kv, 127mAs, FoV 225mm, matrix 512x512, slice thickness 5mm), a CT angiography (120Kv, 663mAs, FoV 261mm, matrix 512x512, slice thickness 0,6mm), and a CTP. For CTP acquisition, a total of fifty milliliters of nonionic iodinated contrast was administered intravenously at 5 mL/s by using a power injector, followed by a saline flush of 20ml at an injection rate of 2ml/s. CTP imaging parameters were 80 kV(peak), 250 mAs, 1.5-second rotation, FoV 18mm, matrix 512x512, and 2-mm thickness (49 slices in total). The follow-up MRI included diffusion-weighted images (DWI, parameters: Repetition time (TR)/echo time (TE) 10800/89ms, matrix 192x192, Field of View (FoV) 240mm, slice thickness 3mm, directions x,y,z, b-values: 0 and 1000 mm/s2) and Gradient-Echo T2*-weighted (GRE: TR/TE 764/26 ms; matrix 384x512 ; FoV 240 mm ; slice thickness 5 mm) sequences.

Supplementary Figure S1

Flow chart of the study.

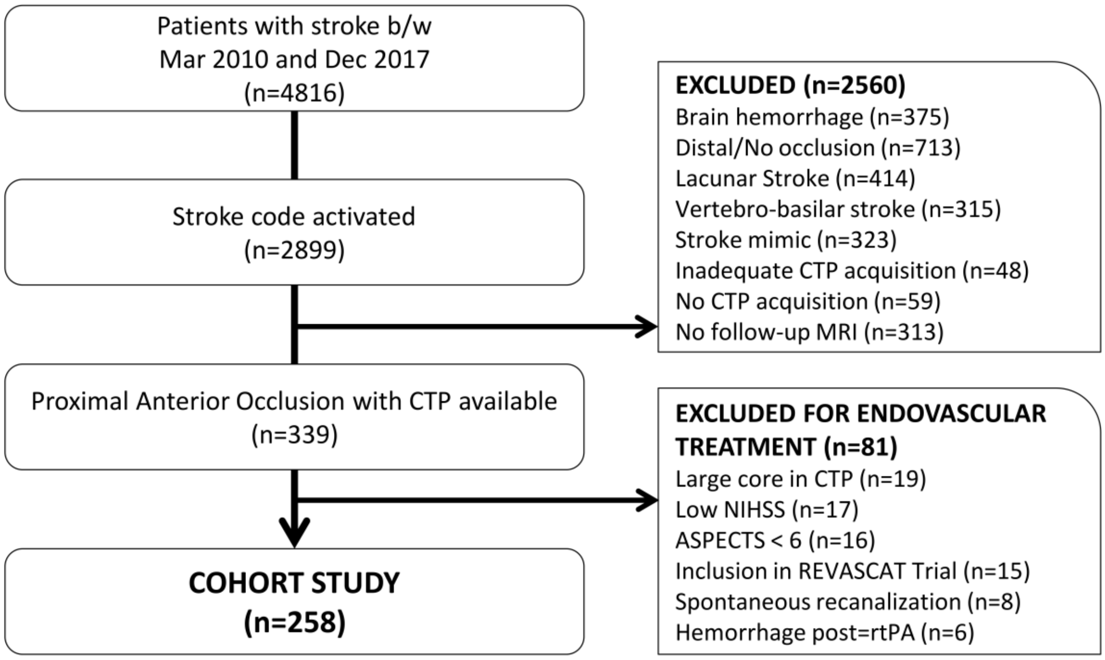

Supplementary Figure S2

Distribution of pretreatment glucose concentration and Very Low CBV regions.

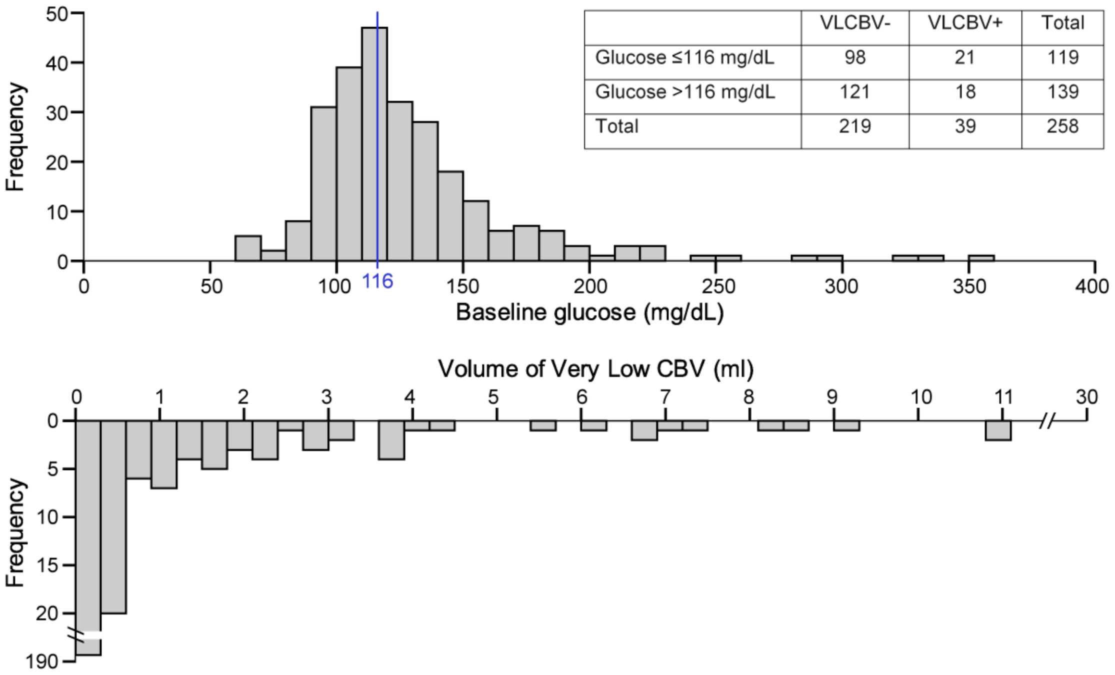

	VLCBV-	VLCBV+	Total
Glucose ≤116 mg/dL	98	21	119
Glucose >116 mg/dL	121	18	139
Total	219	39	258

Figure legend: Histograms showing the distribution of pretreatment glucose levels, the volume of regions with very low cerebral blood volume (VLCBV) and the number of patients in categories defined by the presence of VLCBV regions and glucose levels higher or lower than 116 mg/dL.

Supplementary Table 1

Demographics, baseline and procedure related variables according to the presence of Very Low Cerebral Blood Volume regions.

	**VLCBV - N=219**	**VLCBV + N=39**	**p**
Age (years), median (IQR)	72 (61-80)	67 (56-81)	0.216
Males, n (%)	102 (47)	30 (77)	<0.001
Hypertension, n (%)	123 (56)	24 (62)	0.532
Diabetes, n (%)	34 (16)	2 (5)	0.084
Dyslipidemia, n (%)	83 (38)	15 (39)	0.947
Atrial Fibrillation, n (%)	62 (28)	13 (33)	0.524
Previous Antithrombotic treatment, n (%)	85 (39)	19 (49)	0.245
Baseline SBP (mmHg), median (IQR)	142 (125-160)	135 (125-150)	0.178
Glucose (mg/dL), median (IQR)	120 (106-142)	116 (93-134)	0.088
NIHSS at admission, median (IQR)	16 (10-20)	18 (15-21)	0.049
Ischemic core on CTP (mL), median (IQR)	15 (7-27)	54 (43-72)	<0.001
Hypoperfused tissue on CTP (mL), median (IQR)	129 (87-169)	203 (153-234)	<0.001
Good collaterals, n (%)	166 (76)	19 (49)	0.001
Alteplase + MT, n (%)	105 (48)	12 (31)	0.047
Recanalization (yes), n (%)	174 (80)	30 (77)	0.721
Time to recanalization (min), median (IQR)	291 (205-395)	288 (210-405)	0.851
Recanalization groups			0.922
Recanalization <4.5h, n (%)	78 (36)	14 (36)	
Recanalization >4.5h, n (%)	96 (44)	16 (41)	
No rec, n (%)	45 (21)	9 (23)	
Time to MRI (hours), md (IQR)	40 (26-67)	41 (27-70)	0.971
Cardioembolic origin, n (%)	107 (49)	23 (59)	0.244
Location of the occlusion			0.336
Tandem occlusions, n (%)	31 (14)	9 (23)	
ICA-T or M1, n (%)	171 (78)	28 (72)	
M2, n (%)	17 (8)	2 (5)	

ASPECTS: Alberta Stroke Program Early CT Score; CTP: computed tomographic perfusion; ET: endovascular therapy; ICA-T: internal carotid artery; MT: Mechanical Thrombectomy; NIHSS: National Institutes of Health Stroke Scale; SBP: systolic blood pressure; VLCBV: very low cerebral blood volume.

Supplementary Table 2

Demographics, baseline and procedure related variables according to the occurrence of parenchymal hematoma in patients with Very Low Cerebral Blood Volume regions.

	**No PH N=26**	**PH N=13**	**p**
Age (years), median (IQR)	68 (53-81)	63 (57-76)	0.918
Males, n (%)	20 (77)	10 (77)	1.000
Hypertension, n (%)	12 (46)	3 (23)	0.163
Diabetes, n (%)	1 (4)	1 (8)	1.000
Dyslipidemia, n (%)	10 (39)	5 (39)	1.000
Atrial Fibrillation, n (%)	7 (27)	6 (46)	0.290
Previous Antithrombotic treatment, n (%)	10 (39)	9 (69)	0.070
Baseline SBP (mmHg), median (IQR)	135 (125-149)	135 (125-155)	0.965
Glucose (mg/dL), median (IQR)	109 (91-127)	133 (116-150)	0.023
NIHSS at admission, median (IQR)	18 (14-19)	21 (19-23)	0.010
Ischemic core on CTP (mL), median (IQR)	57 (48-72)	53 (32-65)	0.308
Hypoperfused tissue on CTP (mL), median (IQR)	204 (164-229)	198 (143-264)	0.872
VLCBV (mL), median (IQR)	3.8 (2.3-7.3)	3.0 (2.2-7.1)	0.941
Good collaterals, n (%)	14 (54)	5 (39)	0.365
Alteplase + MT, n (%)	19 (73)	8 (62)	0.486
Recanalization (yes), n (%)	20 (77)	10 (77)	1.000
Time to recanalization (min), median (IQR)	270 (216-373)	360 (290-435)	0.087
Recanalization groups			0.117
Recanalization <4.5h, n (%)	12 (46)	2 (15)	
Recanalization >4.5h, n (%)	8 (31)	8 (62)	
No rec, n (%)	6 (23)	3 (23)	
Time to MRI (hours), md (IQR)	41 (28-70)	41 (26-75)	0.987
Cardioembolic origin, n (%)	15 (58)	8 (62)	0.818
Location of the occlusion			0.663
Tandem occlusions, n (%)	7 (27)	2 (15)	
ICA-T or M1, n (%)	18 (69)	10 (77)	
M2, n (%)	1 (4)	1 (8)	

ASPECTS: Alberta Stroke Program Early CT Score; CTP: computed tomographic perfusion; ET: endovascular therapy; ICA-T: internal carotid artery; MT: Mechanical Thrombectomy; NIHSS: National Institutes of Health Stroke Scale; SBP: systolic blood pressure; VLCBV: very low cerebral blood volume.

Supplementary Table 3

Adjusted multivariate models for the prediction of parenchymal hematoma in the whole population and in subgroups defined by the presence of VLCBV regions.

	**Whole population**	**VLCBV -**	**VLCBV +**
	OR 95%CI<br>p value	OR 95%CI<br>p value	OR 95%CI<br>p value
**Pretreatment glucose (per IQR)**	0.97 (0.71-1.32)<br>p=0.834	0.80 (0.55-1.16)<br>p=0.244	3.15 (1.08-9.19)<br>p=0.036
**Baseline NIHSS (per IQR)**	1.42 (1.02-1.96)<br>p=0.037	1.18 (0.81-1.70)<br>p=0.394	2.23 (0.71-7.29)<br>p=0.165
**Recanalization >4'5h from stroke onset (vs <4'5h)**	5.24 (2.03-13.51)<br>p=0.001	1.18 (0.81-1.70)<br>p=0.394	18.0 (1.28-252.70)<br>p=0.032
**No recanalization (vs <4'5h)**	2.51 (0.81-7.80)<br>p=0.112	2.38 (0.60-9.47)<br>p=0.220	2.51 (0.16-40.10)<br>p=0.516
**Cardioembolic etiology (vs no)**	2.12 (1-04-4.32)<br>p=0.039	2.10 (0.90-4.87)<br>p=0.085	0.94 (0.11-7.94)<br>p=0.951

VLCBV: Very low cerebral blood volume; NIHSS: National Institutes of Health Stroke Scale; PH: parenchymal hematoma. The Hosmer–Lemeshow test showed an adequate goodness-of-fit of the three models (p>0.05 for all).
